# Anti-Inflammatory, Antioxidant, Moisturizing, and Antimelanogenesis Effects of Quercetin 3-O-β-D-Glucuronide in Human Keratinocytes and Melanoma Cells via Activation of NF-κB and AP-1 Pathways

**DOI:** 10.3390/ijms23010433

**Published:** 2021-12-31

**Authors:** Anh Thu Ha, Laily Rahmawati, Long You, Mohammad Amjad Hossain, Jong-Hoon Kim, Jae Youl Cho

**Affiliations:** 1 Department of Integrative Biotechnology, Sungkyunkwan University, Suwon 16419, Korea; anhthu95.vn@gmail.com (A.T.H.); lyrahma0106@g.skku.edu (L.R.); youlonghc@gmail.com (L.Y.); 2 Department of Veterinary Physiology, College of Medicine, Chonbuk National University, Iksan 54596, Korea; mamjadh2@gmail.com; 3 Biomedical Institute for Convergence at SKKU (BICS), Sungkyunkwan University, Suwon 16419, Korea

**Keywords:** quercetin 3-O-β-D-glucuronide, UV protection, moisturizing, antimelanogenesis, AP-1, NF-κB

## Abstract

Quercetin 3-O-β-D-glucuronide (Q-3-G), the glucuronide conjugate of quercetin, has been reported as having anti-inflammatory properties in the lipopolysaccharide-stimulated macrophages, as well as anticancer and antioxidant properties. Unlike quercetin, which has been extensively described to possess a wide range of pharmacological activities including skin protective effects, the pharmacological benefits and mechanisms Q-3-G in the skin remained to be elucidated. This study focused on characterizing the skin protective properties, including anti-inflammatory and antioxidant properties, of Q-3-G against UVB-induced or H_2_O_2_-induced oxidative stress, the hydration effects, and antimelanogenesis activities using human keratinocytes (HaCaT) and melanoma (B16F10) cells. Q-3-G down-regulated the expression of the pro-inflammatory gene and cytokine such as *cyclooxygenase-2* (*COX-2*) and *tumor necrosis factor (TNF)-α* in H_2_O_2_ or UVB-irradiated HaCaT cells. We also showed that Q-3-G exhibits an antioxidant effect using free radical scavenging assays, flow cytometry, and an increased expression of nuclear factor erythroid 2- related factor 2 (Nrf2). Q-3-G reduced melanin production in α-melanocyte-stimulating hormone (α-MSH)-induced B16F10 cells. The hydration effects and mechanisms of Q-3-G were examined by evaluating the moisturizing factor-related genes, such as *transglutaminase-1 (TGM-1)*, *filaggrin (FLG)*, and *hyaluronic acid synthase (HAS)-1*. In addition, Q-3-G increased the phosphorylation of c-Jun, Jun N-terminal kinase (JNK), Mitogen-activated protein kinase (MAPK) kinase 4 (MKK4), and TAK1, involved in the MAPKs/AP-1 pathway, and the phosphorylation of IκBα, IκB kinase (IKK)-α, Akt, and Src, involved in the NF-κB pathway. Taken together, we have demonstrated that Q-3-G exerts anti-inflammatory, antioxidant, moisturizing, and antimelanogenesis properties in human keratinocytes and melanoma cells through NF-κB and AP-1 pathways.

## 1. Introduction

The skin, the largest organ of the body, offers a barrier that plays a major role in protecting the body from the external environment and damaging factors such as ultraviolet radiation, infectious microbes, and harmful chemicals. This barrier also regulates the body’s electrolyte balance and temperature and prevents moisture loss [1,2]. Human skin is composed of three layers: the epidermis, dermis with appendages, and subcutaneous tissue [3]. The epidermal layer plays an important role in maintaining body temperature. Keratinocytes, the abundant component in the skin epidermis, tightly interact with another through desmosomes and tight junctions that allow an effective physicochemical barrier [4]. Although the skin functions as a protective barrier, ultraviolet (UV) radiation has a significant effect on most cells, including keratinocytes, the outer layer of the skin. UV radiation can cause skin aging, skin damage, wrinkles, and hyperpigmentation, which is considered to be one of the most censorious risk factors [5]. UV light consists of three wavelength ranges: UVA (320–400 nm), UVB (280–320 nm), and UVC (200–280 nm). Besides hydrogen peroxide (H_2_O_2_), which has been known to contribute to oxidative stress, UVB can lead to the production of reactive oxygen species (ROS) through the activation of ROS-generating enzymes [6]. The uncontrolled accumulation of ROS in cells including keratinocytes has been described to cause oxidative modifications to lipids, proteins, nucleic acids, and other intracellular molecules that subsequently lead to various types of skin damage and aging-related processes such as cell death, oxidative stress, and inflammation [7,8]. In addition, the oxidative stress response is known to influence the damage of various cellular components [9]. Damaged skin cells can also lead to inflammatory responses, resulting in chronically damaged skin [10]. One type of UVB or H_2_O_2_-mediated skin damage through biological processes is the expression of inflammatory genes such as *cyclooxygenase-2* (*COX-2*) and pro-inflammatory cytokines [11]. Therefore, the development of safer, more robust, and efficient skin protective effects is essential for preventing and blocking diseases associated with UVB or H_2_O_2_-mediated skin damage.

Skin hydration also plays an important role in maintaining its normal function. In particular, the stratum corneum (SC) has a protective effect against water loss [12]. The water content of the stratum corneum participates in skin desquamation and proper maturation, whereas insufficient water in SC promotes the accumulation and dysfunction of corneocytes [12,13]. At the final stages of keratinocyte differentiation, its plasma membrane and cellular organelles disappear, and then calcium influx induces transglutaminase (TGM) for crosslinking with other proteins. A deficiency or loss of TGM-1 leads to defective crosslinking of the cell envelopment. Lamella ichthyosis is an example of a disease caused by a loss of TGM-1. Furthermore, filaggrin (FLG), the formed keratin matrix, plays a role in SC formation as it acts as a scaffold to bind cornified-envelope proteins and lipids [14] by catalyzing g-glutamyl lysine cross-linking reactions, TGMs, and membrane-associated enzymes, imparting integrity to the SCs [15]. In addition, hyaluronic acid (HA) is a type of natural moisture factor (NMF) involved in moisture retention in the skin and exhibits a distinct profile in intrinsic skin aging, is a drug delivery agent, and is an important component of the extracellular matrix [16,17]. HA plays a role in increasing skin moisture by regulating the hyaluronic acid synthase (HAS) genes; besides, retinoic acid (vitamin A) can effectively regulate HA in the epidermis [17]. TGM-1, FLG, and HAS-1,2,3 are genes associated with NMF production [18]. Moreover, the activation of transcription factors such as activator protein-1 (AP-1) and nuclear factor (NF)-κB are involved in the production of pro-inflammatory substances that optimize the skin barrier and skin hydration through the AP-1 and NF-κB pathways [19]. The upstream signaling enzymes in the AP-1 pathway include mitogen-activated protein kinases (MAPKs) such as c-Jun N-terminal kinase (JNK), extracellular signal-regulated kinase (ERK), and p38, while Src, Akt, IκBα-kinase (IKKα/β), and κBα (IκBα) belong to the NF-κB pathway [20]. The skin also produces melanin in the epidermal layer, especially in the melanocytes—the major source of melanin—which contribute to the determination of skin color and melanogenesis [21]. UVB irradiation and α-melanocyte-stimulating hormone (α-MSH) can stimulate melanin secretion, thereby triggering melanogenesis [22]. During melanogenesis, α-MSH activates protein kinase A (PKA), protein microphthalmia-associated transcription factor (MITF), and cAMP response element-binding (CREB) [23]. One of the functions of melanin is considered to be the prevention skin damage by UV, including in sunlight. However, the excess production of melanin or the absence of melanin content caused by aging, melanocyte-stimulating hormone, and UV exposure promotes freckles, melasma, senile lentigines, and other hyper/hypopigmentation disorders that could have a negative impact on appearance and skin health [24]. Thus, controlling melanogenesis is also desired to maintain both the health and cosmetic appearance of the human body.

**Figure 1:**
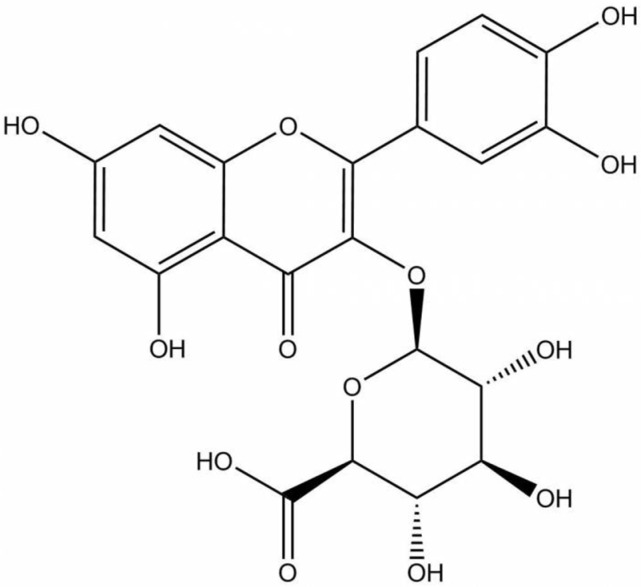
Chemical structure of Q-3-G.

Quercetin is a predominant allocated flavonol-type flavonoid present in vegetables, fruits, beverages, and medicinal plants [25]. Consequently, quercetin has been extensively studied to possess pharmacological benefits including anti-inflammatory, anti-atherosclerotic, antioxidant, anticancer, and skin protective properties and is related to melanogenesis [24,26,27,28,29]. However, several works using sensitive and reliable methods have demonstrated that quercetin has not been detected in plasma [30,31,32,33] and is hardly found in the brain [34,35]. Quercetin, which generally presents as a glycoside form, is well absorbed and mostly hydrolyzed and metabolized in the small intestine, colon, liver, and kidney into conjugated metabolites [30,36,37,38]. Only the conjugated metabolites such as glucuronide or sulfate (methylated or unmethylated, respectively), rather than quercetin aglycone or its glycosides, are detected in the circulating blood. 3′-methyl-quercetin-3-glucuronide, quercetin-3-glucuronide, and quercetin-3’-sulfate have been identified as major plasma metabolites after 1.5 h from the consumption of fried onions [30,32]. These quercetin metabolites are formed in the small intestine and liver by biotransformation enzymes including UDP-glucuronyltransferases as a result of phase II metabolism [29,39,40]. It was also reported that conjugated metabolites of quercetin accumulate in human plasma in the concentration range of 10^−7^ to 10^−6^ M after the periodic ingestion of onions with meals for 1 week [41]. The half-life of quercetin metabolites is quite high, at about 11 to 28 h [29]. Since these compounds have been observed as the main forms of conjugated quercetin metabolites, studies focusing on the biological activity of flavonols using these conjugated metabolites would be essential, which prompted us to study the biological activity of one of the conjugated quercetin metabolites. One of the most abundant glucuronide metabolites found mainly in plasma and tissues including the liver, kidney, and brain is quercetin 3-O-β-D-glucuronide (Q-3-G) (Figure 1) [31,35,40,42,43], which may generate similar, stronger, or weaker activities compared with parent compounds in different models. Q-3-G is transported to target tissues via plasma to exert its biological activity [39]. Q-3-G has also been shown to be an effective metabolite in rat plasma after oral administration of quercetin [43]. In other studies, Q-3-G-containing metabolite was found to be accumulated in the aorta after the administration of quercetin-rich food and has been identified as an active principal detected in human blood ranging from 0.1–10 μM [44]. Additionally, Q-3-G can be found in wine [45] and in medicinal plants such as *Hypericum hirsutum*, *Nelumbo nucifera*, *Oenothera biennis*, and green beans [46,47,48,49]. Q-3-G has been described as an anti-inflammatory component under LPS-stimulated conditions and showed antioxidant properties in blood plasma [50,51,52]. However, compared with the long history of application and wide study of quercetin, the study of Q-3-G remains to be explored. Therefore, Q-3-G was chosen as a representative of quercetin metabolites as an active in vivo player of quercetin. In addition, the role of Q-3-G on skin protective effects has also not been fully elucidated yet. In this study, using various in vitro assessments, we investigated the skin protective effects of Q-3-G against UVB or H_2_O_2_-induced oxidative stress and inflammation, the skin hydration in HaCaT (a human keratinocyte cell line) cells, and antimelanogenesis activity in B16F10 (a murine melanoma cell line) cells.

## 2. Results

### 2.1. Anti-Inflammatory and Antioxidant Effects of Q-3-G against UVB or H_2_O_2_-Stimulated HaCaT Cells

**Figure 2:**
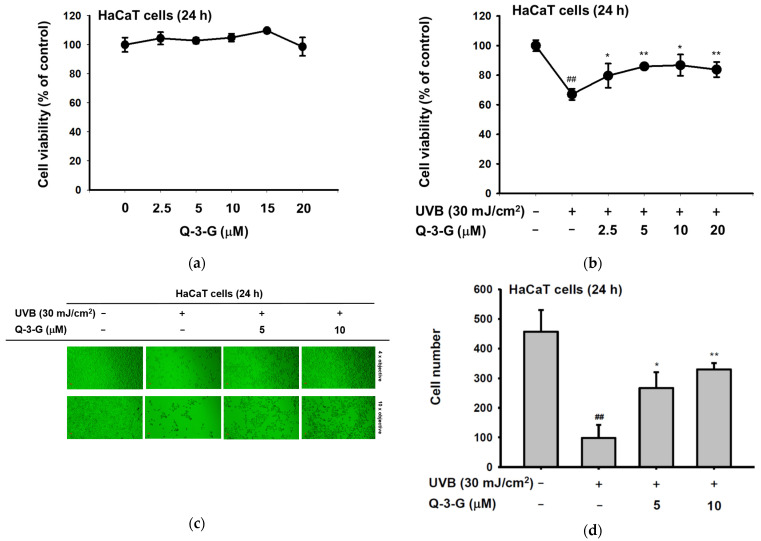

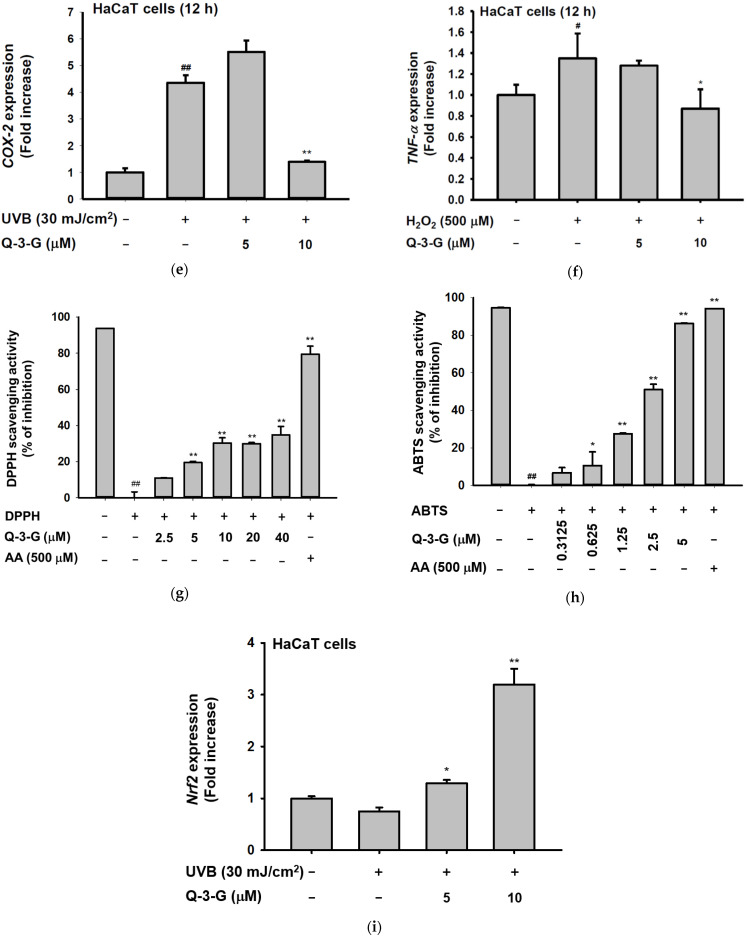

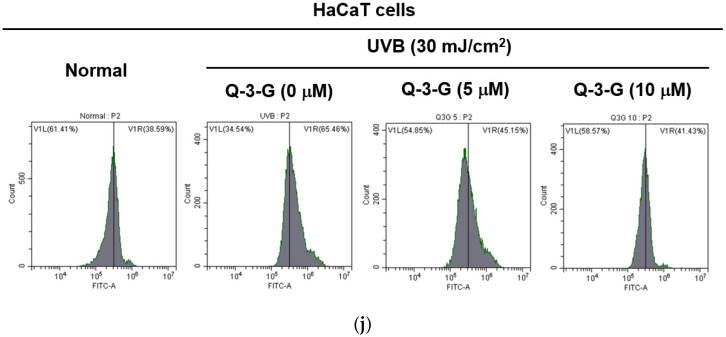
Anti-inflammatory and antioxidant effects of Q-3-G. (**a**) Treatment of HaCaT cells with various concentrations of Q-3-G (0–20 μM) for 24 h, using a conventional MTT assay to determine cell viability. (**b**) HaCaT cells irradiated with UVB (30 mJ/cm^3^) for 10 s in the absence or presence of Q-3-G (0–20 μM), followed by incubation for 24 h. A conventional MTT assay was used to determine cell viability. (**c**,**d**) HaCaT cells were treated with Q-3-G (5, 10 μM) and UVB irradiation (30 mJ/cm^3^) for 24 h. The representative cell morphology of Q-3-G against UVB was observed via microscopy, and the number of cells was then plotted. (**e**,**f**) Anti-inflammatory effect of Q-3-G in HaCaT cells. The HaCaT cells were irradiated with UVB (30 mJ/cm^3^) for 10 s or incubated with H_2_O_2_ (500 μM) with or without Q-3-G (5–10 μM) and further incubated for 12 h. The mRNA expression levels of *COX-2* (**e**) and *TNF-α* (**f**) were determined by quantitative real-time PCR. *GAPDH* was used as a housekeeping gene. (**g**–**j**) Antioxidant effect of Q-3-G in HaCaT cells. Q-3-G (0–40 μM) was reacted with 2,2-diphenylpicrylhydrazyl (DPPH) in the dark at 37 °C for 30 min. The absorbance at 517 nm was measured spectrophotometrically (**g**). Q-3-G was reacted with 2,2-azinobis-(3-ethylbenzothiazoline-6-sulfonic acid (ABTS) at different concentrations in the dark at 37 °C for 30 min. The absorbance at 730 nm was measured spectrophotometrically (**h**). Ascorbic acid was used as a control substance. (**i**) The HaCaT cells were irradiated with UVB (30 mJ/cm^3^) for 10 s with or without Q-3-G (5–10 μM) and after incubating for 12 h, the mRNA level of Nrf2 was determined by quantitative real-time PCR. *GAPDH* was used as a housekeeping gene. (**j**) The representative result of intracellular ROS production was measured by flow cytometry using DCFDA staining in HaCaT cells with or without UVB irradiation (30 mJ/cm^3^) and Q-3-G (0-10 μM) were treated for 24 h. Results (**a**,**b**,**d**–**i**) are expressed as mean ± SD. # *p* < 0.05 and ## *p* < 0.01 compared to normal group (no treatment), and * *p* < 0.05 and ** *p* < 0.01 compared to inducer alone (UVB, H_2_O_2_, DPPH, or ABTS).

Skin tissue can be damaged by UV radiation. UV radiation activates several harmful factors, including oxidative stress, apoptotic cell death, inflammation, and skin aging. In order to determine the UV protective activities of Q-3-G, initially, we evaluated whether Q-3-G has any effects on the cell viability of human keratinocytes. Using a 3-(4-5-Dimethylthiazol-2-yl)-2,5-diphenyl-243 tetrazolium bromide (MTT) assay, we investigated the cytotoxic effects of Q-3-G in HaCaT cells. As shown in Figure 2a, there was no significant interference on cell viability at a concentration of 2.5–20 μM by Q-3-G, which indicated that Q-3-G showed no cytotoxicity up to 20 μM and did not harm the keratinocytes, the superficial layer of the skin cells. Next, we evaluated the skin-protective effects of Q-3-G in HaCaT cells exposed to UVB. After UVB irradiation at 30 mJ/cm^2^, Q-3-G significantly protected HaCaT cells from UVB-induced cell death in a concentration-dependent manner (Figure 2b). In agreement with MTT assay, using a camera connected to a microscope to capture the morphology of HaCaT cells, Q-3-G protected against cell death due to UVB-irradiated HaCaT cells up to a concentration of 10 μM (Figure 2c). The number of HaCaT cells under each condition was 482 for the untreated group, 91 for the UVB 30 mJ/cm^2^ group, 276 for the UVB 30 mJ/cm^2^ plus 5 μM Q-3-G group, and 320 for the UVB 30 mJ/cm^2^ plus 10 μM Q-3-G group (Figure 2d). These results confirmed that Q-3-G inhibits UVB-induced HaCaT damage and rescues the skin-protective effect from UVB irradiation.

The occurrence and exacerbation of inflammation in the body occasionally activate macrophages and release some cytokines. Inflammation is a hallmark of oxidative stress. We investigated the anti-inflammatory effect of Q-3-G against UVB or H_2_O_2_-induced inflammation. It is well known that UVB can promote DNA damage and inflammation in skin cells [10]. We irradiated the HaCaT cells with UVB 30mJ/cm^2^ and incubated the HaCaT cells with H_2_O_2_ (500 μM). We employed a real-time PCR assay to check the mRNA expression of inflammatory enzyme *COX-2* and pro-inflammatory cytokine *TNF-α*. As shown by the results in Figure 2e,f, a 10 μM concentration of Q-3-G significantly inhibited the mRNA expression of *COX-2* and *TNF-α*. Consequently, Q-3-G suppressed the inflammatory responses induced in HaCaT cells after exposure to UVB irradiation or H_2_O_2_.

We used 2,2′-azinobis-(3-ethylbenzothiazoline-6-sulfonic acid) (ABTS) and 2,2-diphenylpicrylhydrazyl (DPPH) radical scavenging assays—widely used model systems to investigate the scavenging activities in vitro of natural compounds—to assess the antioxidant property of Q-3-G at different concentrations. HaCaT cells were treated with Q-3-G in a concentration-dependent manner from 2.5–40 μM in the presence of DPPH, and the result showed a significant scavenging activity at more than 2.5 μM (Figure 2g). The ABTS assay also significantly demonstrated effects of Q-3-G on free radical scavenging in concentration-dependent manners with IC_50_ value of 1.75 μM (Figure 2h). Ascorbic acid (AA), as a positive control, showed the greatest antioxidant effects in both DPPH and ABTS assays. Nuclear factor erythroid 2-related factor 2 (Nrf2), one of the antioxidant response element (ARE)-dependent genes, has been reported to regulate the protective oxidoreductases and its nucleophilic substrates, thereby promoting antioxidant enzymes to remove damage and repair systems [53]. Next, we examined the expression level of Nrf2 in UVB-irradiated HaCaT cells. Strengthening the above findings, Q-3-G significantly enhanced the expression level of Nrf2 upon UVB irradiation compared to the control in a concentration-dependent manner, up to 10 μM (Figure 2i), which implied that Q-3-G presents an antioxidant effect both chemically and biologically with a free radical scavenging property and increases the protective effect of the Nrf2-dependent ARE signaling, respectively. Furthermore, we also performed ROS flow cytometry using dichlorodihydrofluorescein diacetate (DCFDA) in the UV condition. The antioxidant is widely known to suppress cellular stressor by inhibiting ROS production [54]. The result showed that treatment with Q-3-G at concentrations of 5 and 10 μM decreased the level of UVB-induced ROS (Figure 2j). Taken together, these data implied that Q-3-G exerts skin-protective properties such as anti-inflammatory and antioxidant effects against H_2_O_2_ or UVB-induced oxidative stress and inflammation in HaCaT cells.

### 2.2. Antimelanogenesis Effect in B16F10 and Moisturizing Effect in HaCaT Cells of Q-3-G

**Figure 3:**
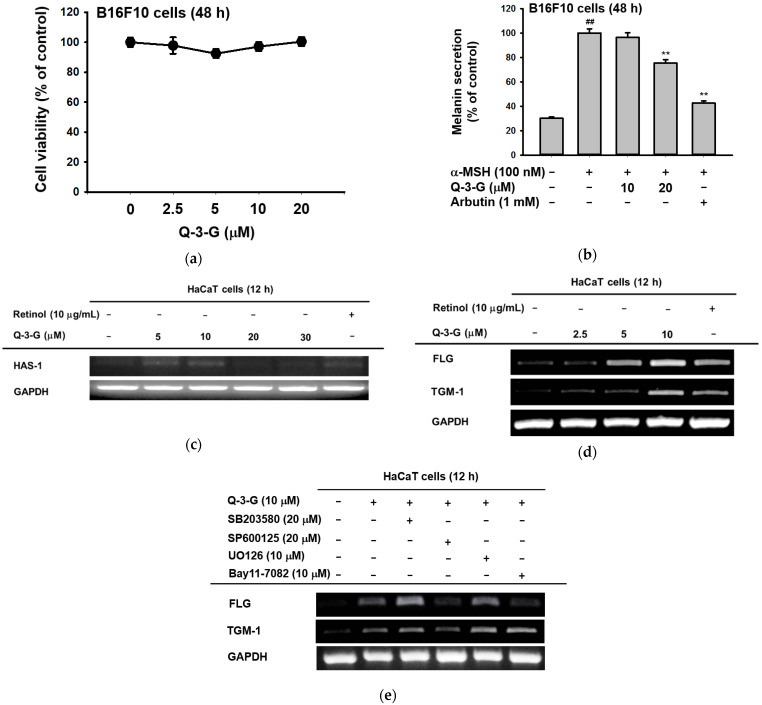
Antimelanogenesis and moisturizing effects of Q-3-G. (**a**) Treatment of B16F10 cells with various concentrations of Q-3-G (0–20 μM) for 48 h, using a conventional MTT assay to determine cell viability. (**b**) B16F10 cells were treated with Q-3-G (10–20 μM) or arbutin (1 mM) for 48 h, and melanin secretion and intracellular melanin were measured at 475 nm. Results are expressed as mean ± SD. ## *p* < 0.01 compared to normal group (no treatment), and * *p* < 0.05 and ** *p* < 0.01 compared to inducer alone (α-MSH). (**c**) The expression level of HAS-1 was measured by RT-PCR in HaCaT cells treated with Q-3-G (5–30 μM) and retinol (10 μg/mL) for 12 h. (**d**) The transcriptional levels of FLG and TGM-1 were determined by RT-PCR in HaCaT cells treated with Q-3-G (2.5–10 μM) and retinol (10 μg/mL) for 12 h. (**e**) MAPK inhibitor (SB203590, SP600125, UO126) and NF-κB inhibitor (Bay117082) were treated with HaCaT cells for 30 min and incubated with Q-3-G for 12 h. The mRNA expression levels of *FLG, TGM-1* and *GAPDH* were determined by RT-PCR. Results (**a**,**b**) are expressed as mean ± SD. ## *p* < 0.01 compared to normal group (no treatment), and ** *p* < 0.01 compared to inducer alone (α-MSH).

To examine the effects of Q-3-G on melanogenesis, the melanoma cells were treated with α-MSH for 48 h to secrete melanin in B16F10 cells. By employing MTT assays, we first confirmed that Q-3-G had no cytotoxicity in B16F10 cells at concentrations up to 20 μM for 48 h (Figure 3a). This result showed, for the next assessments that the effects of Q-3-G in B16F10 were not due to cell death. Next, we evaluated B16F10 with Q-3-G and α-MSH for 48 h to determine the effects of Q-3-G on melanogenesis. As shown in Figure 3b, Q-3-G significantly inhibited melanin secretion. The positive control arbutin also significantly decreased melanin secretion.

The skin moisture-related molecule is hyaluronic acid (HA). As previously mentioned, HA, distributed throughout the epidermis and dermis, is an NMF that enhances skin hydration [55]. We used a reverse transcription PCR (RT-PCR) assay to examine the transcription level of *HAS-1* after 12 h with a concentration of Q-3-G of up to 30 μM. HAS promotes the accumulation of intermediate-sized HA within keratinocytes [12]. As shown in Figure 3c, the expression of *HAS-1* was increased by treatment with Q-3-G up to 10 μM. In addition to the moisturizing factor, we also examined the expression of *FLG* and *TGM-1*. We used retinol as a positive control group. The transcription levels of *FLG* and *TGM-1* increased notably after treatment with Q-3-G at concentrations up to 10 μM (Figure 3d). These findings suggest that Q-3-G has skin moisturizing activity in HaCaT cells. Moreover, using SB203580 (p38 inhibitor), SP600125 (JNK inhibitor), UO126 (ERK inhibitor), and Bay11-7082 (NF-κB inhibitor), we examined the involvement of MAPK and NF-κB signaling pathways in the moisturizing factor of Q-3-G-treated HaCaT cells. Based on Q-3-G-induced gene expression, both the levels of *TGM-1* and *FLG* were reduced by treatment with SP600125, a JNK inhibitor (Figure 3e), suggesting that JNK is required to increase the expression of TGM-1 and FLG in terms of the skin moisturizing effect of Q-3-G. Additionally, FLG expression was also decreased by Bay11-7082, an inhibitor of IKKα and IκBα, suggesting that the pharmaceutical mechanism of Q-3-G also could belong to NF-κB signaling pathway.

### 2.3. Moisturizing-Related Signaling Pathways of Q-3-G via Activation of AP-1 and NF-kB

**Figure 4:**
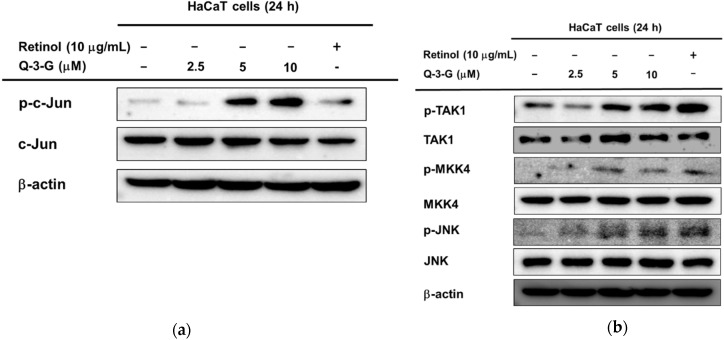

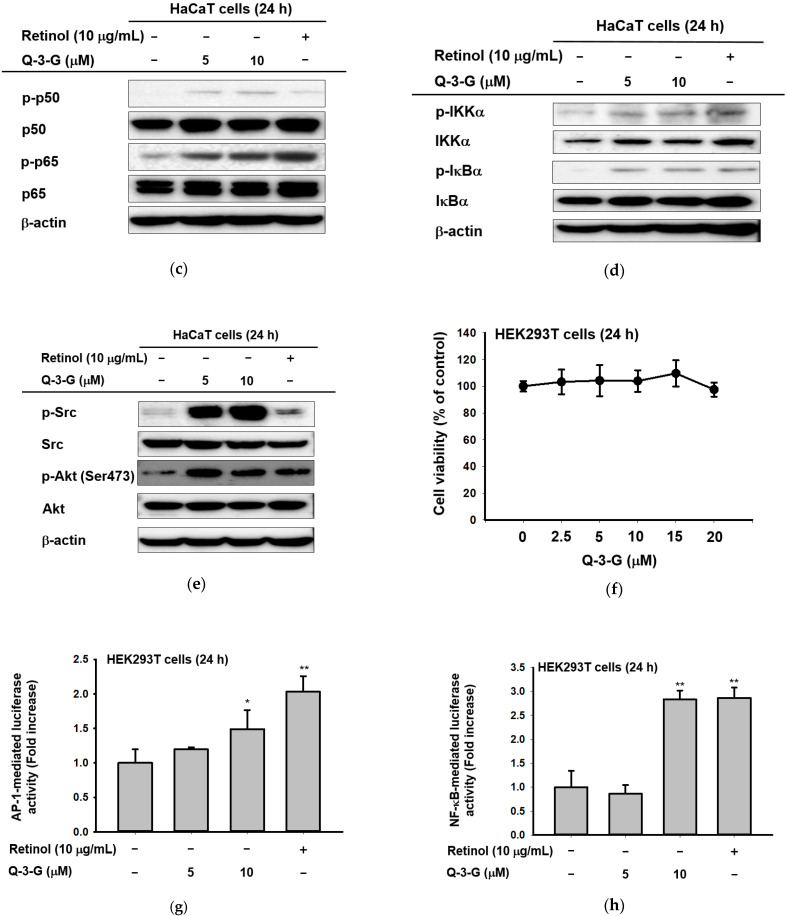
The effects of Q-3-G on AP-1 and the NF-κB signaling pathways. (**a**) HaCaT cells were incubated with Q-3-G (2.5, 5, and 10 μM) and retinol (10 μg/mL) for 24 h, using immunoblot analysis to determine the phosphorylation of c-Jun. (**b**) HaCaT cells were incubated with Q-3-G (2.5, 5, and 10 μM)) and retinol (10 μg/mL) for 24 h, using immunoblot analysis to determine the phosphorylation of TAK1, MKK4, and JNK. (**c**,**d**) The phosphorylation of p50, p65 (**c**), IKKα, and IκBα (**d**) was determined by immunoblot analysis after incubation of HaCaT cells with Q-3-G (5 and 10 μM) and retinol (10 μg/mL) for 24 h. (**e**) The effects of Q-3-G on the phosphorylation of Src and Akt (Ser 473) were measured by immunoblot analysis with Q-3-G (5 to 10 μM) and retinol (10 μg/mL). The expression of β-actin was used as control protein. (**f**) Treatment of HEK293T cells with various concentrations of Q-3-G (0–20 μM) for 24 h, using a conventional MTT assay to determine cell viability. (**g**,**h**) HEK293T cells overexpressing AP-1-luc (**g**) and NF-κB-Luc (**h**) were treated with Q-3-G (5 and 10 μM) and retinol (10 μg/mL) for 24 h. Results (**f**,**g**,**h**) are expressed as mean ± SD. * *p* < 0.05 and ** *p* < 0.01 compared to normal (no treatment).

Based on the previous mRNA results, we hypothesized that the involvement of AP-1 and NF-κB signaling on the hydration effect of Q-3-G can also occur in the protein levels. Using a Western blot assay, we examined the phosphorylation of upstream molecules of AP-1 and NF-κB. First, in the AP-1 pathway, the phosphorylation of c-Jun was increased after incubation of HaCaT cells with Q-3-G for 24 h (Figure 4a). The level of p-JNK was also increased after treatment with Q-3-G at concentrations of 2.5, 5 and 10 μM. The total form of JNK remained unchanged (Figure 4b). We further examined the phosphorylation of MKK4 and TAK-1, which are upstream regulators of JNK. As shown in Figure 3c, after the treatment of HaCaT cells with Q-3-G, the protein levels of p-MKK4 and p-TAK1 were also upregulated.

In addition to NF-κB signaling, Q-3-G increased the phosphorylation of p50 and p65, a subunit of NF-κB (Figure 4c). Moreover, we evaluated the activation of IKKα and IκBα, which also were increased by Q-3-G up to 10 μM compared to the control group (Figure 4d). Eventually, we determined the activation of upstream molecules involved in the NF-κB signaling pathway. The phosphorylation of Src and Akt (Ser473) was enhanced by Q-3-G treatment (Figure 4e). Overall, these data indicate that the skin protection effects of Q-3-G target TAK1 and Src activation by increasing the protein level, thereby upregulating the activity of AP-1 and NF-κB, respectively.

### 2.4. Effects of Q-3-G on the Transcriptional Activation of AP-1 and NF-kB

We investigated the cell cytotoxicity of Q-3-G in human embryonic kidney cell line HEK293T cells by an MTT assay (Figure 4f). The viability of HEK293T cells was not significantly affected after treatment with Q-3-G (2.5–20 μM) compared to untreated cells.

We employed a luciferase assay to determine whether Q-3-G could modulate the NF-κB and AP-1 promoter assays. We confirmed that Q-3-G increased AP-1-mediated luciferase activity (Figure 4g) and NF-κB-mediated luciferase activity (Figure 4h) in a concentration-dependent manner. These results validate that AP-1 activation and NF-κB activation are important pharmacological targets of Q-3-G.

## 3. Discussion

In the previously published studies, Q-3-G has been used to inhibit ear edema, peritoneal permeability, and pulmonary edema [50], acting as a potent antioxidant in blood plasma with low-density lipoprotein [42], decreasing peroxynitrite-induced oxidative modification [51], exerting an anti-inflammatory effect by inhibiting JNK and ERK signaling pathways under LPS challenge [52], and possessing anticancer properties against breast cancer cells [56]. However, unlike its widely studied parent compound, quercetin, the pharmacological benefits and mechanisms of Q-3-G remain to be explored. To the best of our knowledge, the role of Q-3-G on skin protective effects has not been fully elucidated. In this study, using various in vitro assessments, we focused on characterizing the skin protective effects of Q-3-G against UVB or H_2_O_2_-induced oxidative stress and inflammation, skin hydration in HaCaT (a human keratinocyte cell line), and antimelanogenesis activity in B16F10 (a murine melanoma cell line).

The viability of HaCaT, B16F10, and HEK 293T cells was determined after treatment with Q-3-G over a range of non-cytotoxic concentrations. The results showed that there was no significant inhibition of cell viability in HaCaT, B16F10, and HEK 293T cells over concentrations of Q-3-G up to 20 μM (Figure 2a, Figure 3a and Figure 4f). The cell viability in HaCaT, B16F10, and HEK 293T cells at concentrations of Q-3-G up to 20 μM was found to be lower than the concentration of quercetin when it is used to investigate the cytotoxic effects in SCC-9 and HSC-6 cells (50 μM) [57] or sperm viability (50–100 μM) [58]. However, Q-3-G was shown to be much less toxic compared to its aglycone [59]. The lack of a free OH group at the three-position may contribute to the less toxic effect of Q-3-G [29].

UVB rays with a wavelength of 280–315 nm penetrate the upper layer of the skin and are more effective than UVA and UVC [60]. UVB rays are the reason for most skin cancers and cell death. Furthermore, cell damage in HaCaT cells has been shown to be induced by UVB irradiation [61]. Consequently, we investigated the cell viability of HaCaT cells after UVB irradiation compared with Q-3-G treatment. As shown in previous reports, one of the main factors that lead to damaged cells, tissues, and organs is UVB exposure [62,63]. Q-3-G restored the diminished cell viability induced by UVB irradiation (Figure 2b–d). The results are shown to be similar to the previous study on the effect and mechanism on UVB-induced cytotoxicity in HaCaT of quercetin [64].

Several studies have described that UVB can promote severe inflammatory responses, leading to problems in skin [1,2,4,27]. UVB activates important pro-inflammatory enzymes such as COX-2 and cytokines such as TNF-α. COX-2 is an inflammation-associated enzyme that is triggered by pro-inflammatory cytokines and mediators [65], which stimulates the inflammatory response. Because COX-2 produces the inflammatory mediator PGE-2, it induces an inflammatory response [66,67], and TNF-α is the major pro-inflammatory cytokine [68] in the inflammatory responses. These inflammatory responses cause skin damage, which results in skin aging [69]. The mRNA expression levels of the inflammatory enzyme COX-2 and the pro-inflammatory cytokines TNF-α were inhibited by Q-3-G (Figure 2e,f). According to a previously published result, quercetin also decreased the gene expression and production of TNF-α [70]. In regard to antioxidant properties, the DPPH and ABTS radical scavenging assays are a rapid, reliable, and reproducible methods for evaluating the in vitro antioxidant activity of pure compounds and plant extracts [54] and have been used widely in common methods to investigate the scavenging activities of natural compounds or extracts [1,46,71,72]. Q-3-G was found to reduce the reactivity of radicals in both assays and flow cytometry in concentration-dependent manners. These results are in accordance with a previous reported study, in which Q-3-G significantly inhibited the formation of ROS in pheochromocytoma PC-12 cells [34]. Besides acting chemically with an antioxidant effect, we showed that Q-3-G increased the level of Nrf2, one of the important regulators of oxidative stress in cells. Several compounds or natural extracts have been reported to possess antioxidant properties through Nrf2-dependent ARE signaling [46,73,74]. Taken together, these findings indicate that Q-3-G possesses antioxidant activity (Figure 2g–j). Melanosome from from melanin synthesis occurs in intracellular organelles [75,76,77]. Overproduction of melanin can affect skin appearances and hyperpigmentation-related skin diseases. We found that treatment with Q-3-G in α-MSH-induced B16F10 cells inhibited melanin secretion. As in previous works, intracellular melanogenesis is also controlled by quercetin metabolites and some quercetin-3-O-β-D-glucopyranosides [24,78,79].

Maintaining healthy skin requires adequate skin hydration, and NMFs and HA have an important role to play in this process [12]. FLG is a constituent of the skin barrier, so the increased level of FLG expression may be associated with moisturizing [80]. In our study, Q-3-G was found to affect skin moisture retention activity by increasing FLG and TGM-1 (Figure 3c,d). The expression levels of FLG and TGM-1 were blocked by JNK, IKKα, and IκBα inhibitors (SP600125 and Bay11-7082, respectively). We focused on MAPK-related enzymes because our studies aimed to determine the molecular mechanisms by which Q-3-G exerts its skin protective effects. JNK plays an important part in oxidative-stress-induced keratinocyte apoptosis [81]. The phosphorylation of c-Jun, TAK1, MKK4, and JNK was increased by Q-3-G. Additionally, retinol did not significantly increase the phosphorylation of c-Jun, TAK1, MKK4, or JNK, suggesting that there are some differences in the inhibitory characteristics between Q-3-G and retinol in the AP-1 signaling pathway. As in previously published results, the JNK-c-Jun/AP-1 pathway is correlated with the anti-apoptotic effect of quercetin [82]. Compared to the previous study [83], by regulating NF-κB and AP-1 signaling pathways, these pathways were also found to be involved in the underlying mechanism of quercetin. In line with the mechanism of its parent compound, our results show that the NF-kB pathway was also included in the skin protective effect of Q-3-G by the activation of p50, p65, IKKα, IκBα, Akt, and Src after treatment with Q-3-G in HaCaT cells. In addition, Q-3-G upregulated AP-1-mediated luc and NF-κB-luc activity (Figure 4). These findings suggest that MAPK-mediated AP-1 and NF-κB signaling contributed to Q-3-G-mediated skin protective properties.

## 4. Materials and Methods

### 4.1. Materials

Q-3-G was obtained from Sigma Aldrich Co. (St. Louis, MO, USA). HaCaT, HEK293T, and B16F10 cells were purchased from the American Type Culture Collection (Rockville, MD, USA). ABTS diammonium salt, DPPH, ascorbic acid (AA), ABTS, and retinol were purchased from Sigma Chemical Co. (St. Louis, MO, USA). Fetal bovine serum (FBS), Dulbecco’s Modified Eagle Medium (DMEM), and phosphate-buffered saline (PBS) were bought from Gibco (Grand Island, NY, USA). 3-(4-5-Dimethylthiazol-2-yl)-2,5-diphenyltetrazolium bromide (MTT) was purchased from Amresco (Brisbane, Australia). TRIzol and PCR premix were purchased from Bio-D Inc. (Seoul, Korea). The cDNA synthesis kits were bought from Thermo Fisher Scientific (Waltham, MA, USA). Forward and reverse primers for PCR (polymerase chain reaction) were synthesized by Macrogen, Inc. (Seoul, Korea). All the antibodies related to the phosphorylated or total forms of the target protein were bought from Cell Signaling Technology (Beverly, MA, USA).

### 4.2. Cell Cultures

HaCaT and B16F10 cells were cultured in DMEM with 10% FBS and 1% antibiotics (streptomycin and penicillin). HEK293T cells were cultured in DMEM with 5% FBS and 1% antibiotics. These cell lines were incubated in 5% CO_2_ at 37 °C.

### 4.3. Cell Viability Tests

B16F10 cells were seeded at 2 × 10^5^ cells per well in 96-well plates for 24 h, different concentrations of Q-3-G (0–20 μM) were added to them, and then they were incubated for 48 h. HaCaT cells were plated in 96-well plates, and the cells were seeded at 6 × 10^5^ cells/mL. After overnight incubation, the cells were incubated with different concentrations of Q-3-G (0–20 μM) for 24 h. HEK293T cells were seeded at 6 × 10^5^ cells/mL in 96-well-plates for 24 h and then treated with Q-3-G (0–20 μM). Incubated cells were treated with 10 μL/well of MTT solution, and after 3 h were treated with 100 μL of MTT Stopping Solution. Using the conventional MTT assay for measured cell viability [84], the absorbance at 570 nm was measured using a reader (BioTek Instruments, Winooski, VT, USA).

### 4.4. mRNA Analysis by Semi-Quantitative RT-PCR and Quantitative Real-Time PCR

HaCaT cells were treated with Q-3-G (5–30 μM) or retinol (10 μg/mL) for 12 h. RNA was precipitated using TRI reagent as reported previously [85]. A cDNA synthesis kit was used to synthesize the complementary DNA. The RT-PCR was conducted using specific reverse and forward primers. Primers for RT-PCR and real-time PCR are listed in Table 1.

### 4.5. DPPH Assay

A DPPH assay was used to assess the radical scavenging capacity of Q-3-G. Methanol was used to dissolve DPPH to a final concentration of 250 μM. Then, 475 mL of DPPH was reacted with 5 mL of Q-3-G (0–40 μM) or AA (500 mM) at 37 °C for 30 min. The positive control was AA. The determination of the DPPH scavenging effect and calculation were performed as described previously [86].

### 4.6. ABTS Assay

An ABTS assay was performed as previously reported [72]. Briefly, equal volumes of 7.4 mM ABTS solution and 2.4 mM potassium persulfate solution were mixed and kept at room temperature overnight to generate ABTS radical cations. ABTS solutions were added to each well of 96-well plates, with the addition of Q-3-G (0–40 μM) or AA (500 mM) per well. The mixtures were incubated at 37 °C for 30 min, and then their absorbance at 730 nm was measured.

### 4.7. Cellular ROS Assay by Flow Cytometry

To check the formation of intracellular ROS, an oxidation-sensitive dye, 2′,7′-dichlorodihydrofluorescein diacetate (DCFDA), was used. HaCaT cells were exposed to UV, and then the cells were harvested and resuspended with 300 μl PBS with 20 μM DCFDA for 30 min at 37 °C in the dark. The cells were then washed with PBS and the fluorescence was measured at 485/535 nm by a Beckman CytoFLEX Flow Cytometer (Beckman Coulter, Brea, CA, USA).

### 4.8. Western Blot Analysis

HaCaT cells were seeded at a density of 6 × 10^5^ cells/mL. After incubation overnight, different concentrations of Q-3-G (0–10 μM) or retinol (10 μg/mL) were added and further incubated for 24 h. Protein preparation and whole cell lysates were performed as described previously [87]. Specific antibodies were used to detect the total form and phosphorylated form of Jun, JNK, MKK4, TAK1, p50, p65, IKKα, IκBα, Src, and Akt, which were visualized with chemiluminescence reagents [88].

### 4.9. Melanin Content and Secretion Analysis

B16F10 cells were seeded at a density of 2 × 10^5^ cells/mL into 12-well plates and incubated overnight. The culture medium was changed for fresh medium supplemented with Q-3-G (10–20 μM) or arbutin (1 mM). Melanin production was stimulated with a-MSH (100 nM) for 48 h. For the melanin secretion assay, the optical density at 475 nm was measured. Afterwards, the cells were harvested to determine the intracellular melanin content, using a lysis buffer to lyse the cells and pelleting them by centrifugation (12,000 rpm, 5 min). The concentrated cell pellets were dissolved in 100 mL dissolving buffer (1 M NaOH, 10% DMSO) and melted at 55 °C. The absorbance was measured at 405 nm using an optical density reader [89].

### 4.10. UVB Irradiation

HaCaT cells were seeded at a density of 1 × 10^5^ cells/mL into 6-well plates using serum-free MEM and were subjected to 24 h of starvation. HaCaT cells were pre-treated with G-3-Q for 30 min and then UVB irradiation. Then, the HaCaT cells were washed with PBS and exposed to UVB irradiation (UVB Lamp BLX-312, Vilber Lourmat, France). The energy of UVB irradiation was 30 mJ/cm^2^ [90].

### 4.11. H_2_O_2_ Treatment

HaCaT cells were seeded into 6-well plates and subjected to 24 h of starvation using serum-free MEM. The cells were pretreated with different concentrations of Q-3-G (5–10 μM) and after 30 min were incubated with H_2_O_2_ (500 μM) for 12 h.

### 4.12. Luciferase Reporter Gene Assay

HEK 293T cells were seeded at a density of 3 × 10^5^ cells/mL into 24-well plates. The HEK 293T cells were transferred with 2 μg of plasmids containing AP-1-Luc or NF-κB-Luc and b-galactosidase, employing polyethylenimine [91]. After 24 h of incubation, the cells were treated with Q-3-G (5–10 μM) or retinol (10 μg/mL) for a further 24 h. The luciferase assay was performed by a luminometer at absorbance of each sample was measured at 475 nm using a Spectramax 250 microplate reader (Molecular devices, San Jose, CA, USA), as reported previously [92].

### 4.13. Cell Morphology Shooting

HaCaT cells were seeded into 6-well plates at a cell density of 1 × 10^5^ cells/mL. The HaCaT cells were treated with G-3-Q for 30 min and then UVB irradiation. After 24 h, 4×, 10×, and 20× microscope objective photos were taken (Olympus, Tokyo, Japan).

### 4.14. Statistical Analysis

All data are presented as the mean ± standard deviation of at least three independent experiments. The Mann–Whitney test and Student’s t-test were used to compare statistical differences between groups. A *p*-value < 0.05 was regarded as statistically significant. All statistical tests were carried out using SPSS (Ver. 25, SPSS Inc., Chicago, IL, USA).

## 5. Conclusions

**Figure 5:**
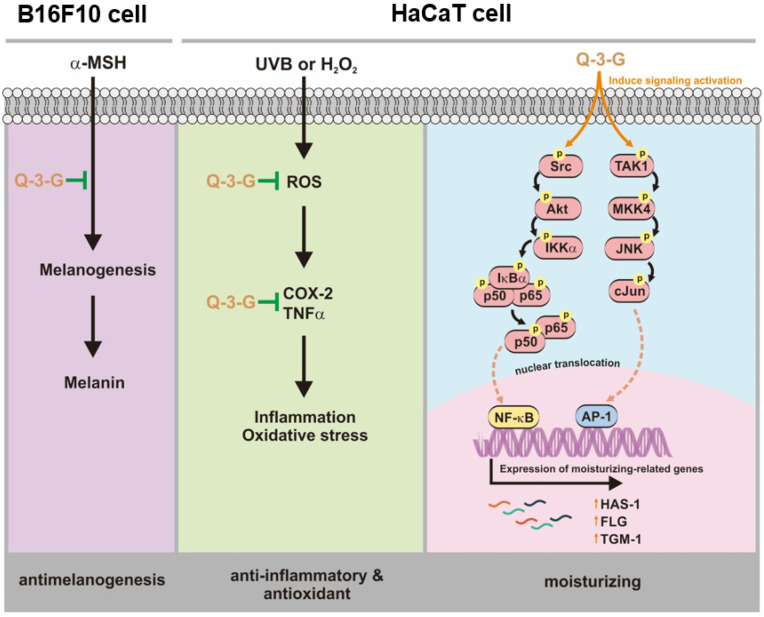
Anti-inflammatory, antioxidant, moisturizing, and antimelanogenesis mechanisms of Q-3-G in human keratinocytes and melanoma cells.

Our research demonstrated that Q-3-G exhibited anti-inflammatory and antioxidant activities to protect against factors of oxidative stress and inflammation under UVB irradiation and H_2_O_2_ exposure. We also demonstrated that Q-3-G has a moisturizer-stimulating ability in HaCaT cells. In addition to the antimelanogenesis effect of Q-3-G, it was shown to inhibit melanin secretion in α-MSH-induced B16F10. Finally, the effect of Q-3-G was mediated through the activation of AP-1 and NF-κB signaling pathways. The skin-protective activities of Q-3-G in HaCaT human keratinocytes cells and B16F10 murine melanoma cells are summarized in Figure 5. This study established that Q-3-G could be effective due to its anti-inflammatory, antioxidant, moisturizing, and antimelanogenesis properties in human keratinocytes and melanoma cells via the activation of NF-κB and AP-1 pathways. In conclusion, the results clearly indicate that this conjugate metabolite has the potential to protect skin cells. Further investigations are needed to validate the effect of Q-3-G in various in vivo models, especially in relation to skin protective models.

## Figures and Tables

**Table 1:** Primer sequences for the analysis of mRNA prepared for RT-PCR and real-time PCR.

Name	Direction	Sequence (5′ to 3′)
Primer Sequences used in RT-PCR		
FLG	Forward	AGGGAAGATCCAAGAGCCCA
	Reverse	ACTCTGGATCCCCTACGCTT
TGM	Forward	CCCCCGCAATGAGATCTACA
	Reverse	ATCCTCATGGTCCACGTACACA
HAS 1	Forward	CCACCCAGTACAGCGTCAAC
	Reverse	CATGGTGCTTCTGTCGCTCT
GAPDH	Forward	GGTCACCAGGGCTGCTTTTA
	Reverse	GATGGCATGGACTGTGGTCA
Primer Sequences used in real-time PCR		
COX 2	Forward	CAGCATTGTAAAGTTGGTGGACTGT
	Reverse	GGGATTTTGGAACGTTGTGAA
TNF α	Forward	CTGCTGCACTTTGGAGTGAT
	Reverse	CCTCTTCTCCTTCCTGATCG
Nrf2	Forward	ACATCCTTTGGAGGCAAGAC
	Reverse	TCGGGTCATTGTGAGTCAGT
GAPDH	Forward	GCGCCCAATACGACCAAATC
	Reverse	GACAGTCAGCCGCATCTTCT

## Data Availability

The data is contained within the article.

## Abbreviations

α-MSH	α-melanocyte-stimulating hormone
AP-1	Activator protein-1
COX-2	Cyclooxygenase-2
CREB	cAMP response element binding
ERK	Extracellular signal-regulated kinase
FLG	Filaggrin
H_2_O_2_	Hydrogen peroxide
HA	Hyaluronic acid
HAS	Hyaluronic acid synthase
JNK	c-Jun N-terminal kinase
LPS	Polysaccharide
MAPKs	Mitogen-activated protein kinases
MITF	Microphthalmia-associated transcription factor
MTT	3-(4-5-Dimethylthiazol-2-yl)-2,5-diphenyl-243 tetrazolium bromide
NMF	Natural moisture factor
PKA	Protein kinase A
Q-3-G	Quercetin 3-O-β-D-glucuronide
ROS	Reactive oxygen species
TGM-1	Transglutaminase-1
UV	Ultraviolet

## References

[1] Kim E., Hwang K., Lee J., Han S.Y., Kim E.-M., Park J., Cho J.Y. (2018). Skin protective effect of epigallocatechin gallate. Int. J. Mol. Sci..

[2] Draelos Z.D. (2012). New treatments for restoring impaired epidermal barrier permeability: Skin barrier repair creams. Clin. Dermatol..

[3] Slominski A.T., Zmijewski M.A., Zbytek B., Tobin D.J., Theoharides T.C., Rivier J. (2013). Key role of CRF in the skin stress response system. Endocr. Rev..

[4] D’Orazio J., Jarrett S., Amaro-Ortiz A., Scott T. (2013). UV radiation and the skin. Int. J. Mol. Sci..

[5] Rittie L., Fisher G.J. (2002). UV-light-induced signal cascades and skin aging. Ageing Res. Rev..

[6] Videira I.F., Moura D.F., Magina S. (2013). Mechanisms regulating melanogenesis. An. Bras. Dermatol..

[7] Che D.N., Xie G.H., Cho B.O., Shin J.Y., Kang H.J., Jang S.I. (2017). Protective effects of grape stem extract against UVB-induced damage in C57BL mice skin. J. Photochem. Photobiol. B.

[8] Jeong D., Lee J., Jeong S.G., Hong Y.H., Yoo S., Han S.Y., Kim J.H., Kim S., Kim J.S., Chung Y.S. (2018). *Artemisia asiatica* ethanol extract exhibits anti-photoaging activity. J. Ethnopharmacol..

[9] McDaniel D., Farris P., Valacchi G. (2018). Atmospheric skin aging-Contributors and inhibitors. J. Cosmet. Dermatol..

[10] Rao C.V., Pal S., Mohammed A., Farooqui M., Doescher M.P., Asch A.S., Yamada H.Y. (2017). Biological effects and epidemiological consequences of arsenic exposure, and reagents that can ameliorate arsenic damage in vivo. Oncotarget.

[11] Sinha K., Das J., Pal P.B., Sil P.C. (2013). Oxidative stress: The mitochondria-dependent and mitochondria-independent pathways of apoptosis. Arch. Toxicol..

[12] Verdier-Sévrain S., Bonté F. (2007). Skin hydration: A review on its molecular mechanisms. J. Cosmet. Dermatol..

[13] Wikramanayake T.C., Stojadinovic O., Tomic-Canic M. (2014). Epidermal differentiation in barrier maintenance and wound healing. Adv. Wound Care.

[14] Sandilands A., Sutherland C., Irvine A.D., McLean W.H. (2009). Filaggrin in the frontline: Role in skin barrier function and disease. J. Cell Sci..

[15] Thacher S.M., Rice R.H. (1985). Keratinocyte-specific transglutaminase of cultured human epidermal cells: Relation to cross-linked envelope formation and terminal differentiation. Cell.

[16] Papakonstantinou E., Roth M., Karakiulakis G. (2012). Hyaluronic acid: A key molecule in skin aging. Dermatoendocrinology.

[17] Brown M.B., Jones S.A. (2005). Hyaluronic acid: A unique topical vehicle for the localized delivery of drugs to the skin. J. Eur. Acad. Dermatol. Venereol..

[18] Kim E., Kim D., Yoo S., Hong Y.H., Han S.Y., Jeong S., Jeong D., Kim J.H., Cho J.Y., Park J. (2018). The skin protective effects of compound K, a metabolite of ginsenoside Rb1 from *Panax ginseng*. J. Ginseng Res..

[19] Lee J.O., Hwang S.H., Shen T., Kim J.H., You L., Hu W., Cho J.Y. (2021). Enhancement of skin barrier and hydration-related molecules by protopanaxatriol in human keratinocytes. J. Ginseng Res..

[20] Augereau P., Patsouris A., Bourbouloux E., Gourmelon C., Abadie Lacourtoisie S., Berton Rigaud D., Soulie P., Frenel J.S., Campone M. (2017). Hormonoresistance in advanced breast cancer: A new revolution in endocrine therapy. Ther. Adv. Med. Oncol..

[21] Lee J.O., Kim E., Kim J.H., Hong Y.H., Kim H.G., Jeong D., Kim J., Kim S.H., Park C., Seo D.B. (2018). Antimelanogenesis and skin-protective activities of *Panax ginseng* calyx ethanol extract. J. Ginseng Res..

[22] Pillai S., Oresajo C., Hayward J. (2005). Ultraviolet radiation and skin aging: Roles of reactive oxygen species, inflammation and protease activation, and strategies for prevention of inflammation-induced matrix degradation—A review. Int. J. Cosmet. Sci..

[23] D’Mello S.A., Finlay G.J., Baguley B.C., Askarian-Amiri M.E. (2016). Signaling pathways in melanogenesis. Int. J. Mol. Sci..

[24] You L., Cho J.Y. (2021). The regulatory role of Korean ginseng in skin cells. J. Ginseng Res..

[25] Sun A.Y., Simonyi A., Sun G.Y. (2002). The “French Paradox” and beyond: Neuroprotective effects of polyphenols. Free Radic. Biol. Med..

[26] Kook D., Wolf A.H., Yu A.L., Neubauer A.S., Priglinger S.G., Kampik A., Welge-Lüssen U.C. (2008). The protective effect of quercetin against oxidative stress in the human RPE in vitro. Investig. Ophthalmol. Vis. Sci..

[27] Park J.M., Cho J.-K., Mok J.Y., Jeon I.H., Kim H.S., Kang H.J., Jang S.I. (2012). Protective effect of astragalin and quercetin on ultraviolet (UV)-irradiated damage in HaCaT cells and Balb/c mice. J. Korean Soc. Appl. Biol. Chem..

[28] Alizadeh S.R., Ebrahimzadeh M.A. (2021). O-Glycoside quercetin derivatives: Biological activities, mechanisms of action, and structure-activity relationship for drug design, a review. Phytother. Res..

[29] Boots A.W., Haenen G.R., Bast A. (2008). Health effects of quercetin: From antioxidant to nutraceutical. Eur. J. Pharmacol..

[30] Day A.J., Mellon F., Barron D., Sarrazin G., Morgan M.R.A., Williamson G. (2001). Human metabolism of dietary flavonoids: Identification of plasma metabolites of quercetin. Free Radic. Res..

[31] Oh J.H., Kim D., Lee H., Kim G., Park T., Kim M.C., Lee Y.J. (2021). Negligible effect of quercetin in the pharmacokinetics of sulfasalazine in rats and beagles: Metabolic inactivation of the interaction potential of quercetin with BCRP. Pharmaceutics.

[32] Mullen W., Boitier A., Stewart A.J., Crozier A. (2004). Flavonoid metabolites in human plasma and urine after the consumption of red onions: Analysis by liquid chromatography with photodiode array and full scan tandem mass spectrometric detection. J. Chromatogr..

[33] Wittig J., Herderich M., Graefe E.U., Veit M. (2001). Identification of quercetin glucuronides in human plasma by high-performance liquid chromatography-tandem mass spectrometry. J. Chromatogr. B Biomed. Sci. Appl..

[34] Shirai M., Kawai Y., Yamanishi R., Kinoshita T., Chuman H., Terao J., Shirai M., Kawai Y., Yamanishi R., Kinoshita T. (2006). Effect of a conjugated quercetin metabolite, quercetin 3-glucuronide, on lipid hydroperoxide-dependent formation of reactive oxygen species in differentiated PC-12 cells. Free Radic. Res..

[35] Ho L., Ferruzzi M.G., Janle E.M., Wang J., Gong B., Chen T.Y., Lobo J., Cooper B., Wu Q.L., Talcott S.T. (2013). Identification of brain-targeted bioactive dietary quercetin-3-O-glucuronide as a novel intervention for Alzheimer’s disease. FASEB J..

[36] da Silva E.L., Piskula M.K., Yamamoto N., Moon J.H., Terao J. (1998). Quercetin metabolites inhibit copper ion-induced lipid peroxidation in rat plasma. FEBS Lett..

[37] Manach C., Texier O., Régérat F., Agullo G., Demigné C., Rémésy C. (1996). Dietary quercetin is recovered in rat plasma as conjugated derivatives of isorhamnetin and quercetin. Nutr. Biochem..

[38] Day A.J., Bao Y., Morgan M.R., Williamson G. (2000). Conjugation position of quercetin glucuronides and effect on biological activity. Free Radic. Biol. Med..

[39] Ulusoy H.G., Sanlier N. (2020). A minireview of quercetin: From its metabolism to possible mechanisms of its biological activities. Crit. Rev. Food Sci. Nutr..

[40] Docampo-Palacios M.L., Alvarez-Hernández A., Adiji O., Gamiotea-Turro D., Valerino-Diaz A.B., Viegas L.P., Ndukwe I.E., de Fátima Â., Heiss C., Azadi P. (2020). Glucuronidation of methylated quercetin derivatives: Chemical and biochemical approaches. J. Agric. Food Chem..

[41] Moon J.H., Nakata R., Oshima S., Inakuma T., Terao J. (2000). Accumulation of quercetin conjugates in blood plasma after the short-term ingestion of onion by women. Am. J. Physiol. Regul. Integr. Comp. Physiol..

[42] Moon J.H., Tsushida T., Nakahara K., Terao J. (2001). Identification of quercetin 3-O-β-D-glucuronide as an antioxidative metabolite in rat plasma after oral administration of quercetin. Free Radic. Biol. Med..

[43] Yang L.-L., Xiao N., Li X.-W., Fan Y., Alolga R.N., Sun X.-Y., Wang S.-L., Li P., Qi L.-W. (2016). Pharmacokinetic comparison between quercetin and quercetin 3-O-β-glucuronide in rats by UHPLC-MS/MS. Sci. Rep..

[44] Mullen W., Edwards C.A., Crozier A. (2006). Absorption, excretion and metabolite profiling of methyl-, glucuronyl-, glucosyl- and sulpho-conjugates of quercetin in human plasma and urine after ingestion of onions. Br. J. Nutr..

[45] Satué-Gracia M.T., Andrés-Lacueva C., Lamuela-Raventós R.M., Frankel E.N. (1999). Spanish sparkling wines (Cavas) as inhibitors of in vitro human low-density lipoprotein oxidation. J. Agric. Food Chem..

[46] Lee S.Y., Kim C.H., Hwang B.S., Choi K.-M., Yang I.-J., Kim G.-Y., Choi Y.H., Park C., Jeong J.-W. (2020). Protective effects of *Oenothera biennis* against hydrogen peroxide-induced oxidative stress and cell death in skin keratinocytes. Life.

[47] Napoli E., Siracusa L., Ruberto G., Carrubba A., Lazzara S., Speciale A., Cimino F., Saija A., Cristani M. (2018). Phytochemical profiles, phototoxic and antioxidant properties of eleven *Hypericum* species—A comparative study. Phytochemistry.

[48] Kashiwada Y., Aoshima A., Ikeshiro Y., Chen Y.P., Furukawa H., Itoigawa M., Fujioka T., Mihashi K., Cosentino L.M., Morris-Natschke S.L. (2005). Anti-HIV benzylisoquinoline alkaloids and flavonoids from the leaves of *Nelumbo nucifera*, and structure-activity correlations with related alkaloids. Bioorg. Med. Chem..

[49] Plumb G.W., Price K.R., Williamson G. (1999). Antioxidant properties of flavonol glycosides from green beans. Redox Rep..

[50] Fan D., Zhou X., Zhao C., Chen H., Zhao Y., Gong X. (2011). Anti-inflammatory, antiviral and quantitative study of quercetin-3-O-beta-D-glucuronide in *Polygonum perfoliatum* L.. Fitoterapia.

[51] Terao J., Yamaguchi S., Shirai M., Miyoshi M., Moon J.H., Oshima S., Inakuma T., Tsushida T., Kato Y. (2001). Protection by quercetin and quercetin 3-O-beta-D-glucuronide of peroxynitrite-induced antioxidant consumption in human plasma low-density lipoprotein. Free Radic. Res..

[52] Park J.Y., Lim M.S., Kim S.I., Lee H.J., Kim S.S., Kwon Y.S., Chun W. (2016). Quercetin-3-O-beta-D-glucuronide suppresses lipopolysaccharide-induced JNK and ERK phosphorylation in LPS-challenged RAW264.7 cells. Biomol. Ther. (Seoul).

[53] Forman H.J., Davies K.J.A., Ursini F. (2014). How do nutritional antioxidants really work: Nucleophilic tone and para-hormesis versus free radical scavenging in vivo. Free Radic. Biol. Med..

[54] Gupta V.K., Sharma S.K. (2006). Plants as natural antioxidants. Indian J. Nat. Prod. Res..

[55] Tammi R., Ripellino J.A., Margolis R.U., Maibach H.I., Tammi M. (1989). Hyaluronate accumulation in human epidermis treated with retinoic acid in skin organ culture. J. Investig. Dermatol..

[56] Yamazaki S., Miyoshi N., Kawabata K., Yasuda M., Shimoi K. (2014). Quercetin-3-O-glucuronide inhibits noradrenaline-promoted invasion of MDA-MB-231 human breast cancer cells by blocking β₂-adrenergic signaling. Arch. Biochem. Biophys..

[57] Zhao J., Fang Z., Zha Z., Sun Q., Wang H., Sun M., Qiao B. (2019). Quercetin inhibits cell viability, migration and invasion by regulating miR-16/HOXA10 axis in oral cancer. Eur. J. Pharmacol..

[58] Khanduja K.L., Verma A., Bhardwaj A. (2008). Impairment of human sperm motility and viability by quercetin is independent of lipid peroxidation. Andrologia.

[59] Yoshino S., Hara A., Sakakibara H., Kawabata K., Tokumura A., Ishisaka A., Kawai Y., Terao J. (2011). Effect of quercetin and glucuronide metabolites on the monoamine oxidase-A reaction in mouse brain mitochondria. Nutrition.

[60] Mainster M.A. (2006). Violet and blue light blocking intraocular lenses: Photoprotection versus photoreception. Br. J. Ophthalmol..

[61] Schuch A.P., Moreno N.C., Schuch N.J., Menck C.F.M., Garcia C.C.M. (2017). Sunlight damage to cellular DNA: Focus on oxidatively generated lesions. Free Radic. Biol. Med..

[62] Janjetovic Z., Jarrett S.G., Lee E.F., Duprey C., Reiter R.J., Slominski A.T. (2017). Melatonin and its metabolites protect human melanocytes against UVB-induced damage: Involvement of NRF2-mediated pathways. Sci. Rep..

[63] Slominski A.T., Hardeland R., Zmijewski M.A., Slominski R.M., Reiter R.J., Paus R. (2018). Melatonin: A cutaneous perspective on its production, metabolism, and functions. J. Investig. Dermatol..

[64] Zhu X., Li N., Wang Y., Ding L., Chen H., Yu Y., Shi X. (2016). Protective effects of quercetin on UVB irradiation-induced cytotoxicity through ROS clearance in keratinocyte cells. Oncol. Rep..

[65] Chen L., Deng H., Cui H., Fang J., Zuo Z., Deng J., Li Y., Wang X., Zhao L. (2017). Inflammatory responses and inflammation-associated diseases in organs. Oncotarget.

[66] Ricciotti E., FitzGerald G.A. (2011). Prostaglandins and inflammation. Arterioscler. Thromb. Vasc. Biol..

[67] Norregaard R., Kwon T.H., Frokiaer J. (2015). Physiology and pathophysiology of cyclooxygenase-2 and prostaglandin E2 in the kidney. Kidney Res. Clin. Pract..

[68] Schuerwegh A.J., Dombrecht E.J., Stevens W.J., Van Offel J.F., Bridts C.H., De Clerck L.S. (2003). Influence of pro-inflammatory (IL-1 alpha, IL-6, TNF-alpha, IFN-gamma) and anti-inflammatory (IL-4) cytokines on chondrocyte function. Osteoarthritis Cartilage.

[69] Dinarello C.A. (2000). Proinflammatory cytokines. Chest.

[70] Min Y.-D., Choi C.H., Bark H., Son H.-Y., Park H.-H., Lee S., Park J.-W., Park E.-K., Shin H.-I., Kim S.-H. (2007). Quercetin inhibits expression of inflammatory cytokines through attenuation of NF-kappaB and p38 MAPK in HMC-1 human mast cell line. Inflamm. Res..

[71] Om P., Sharma T.K.B. (2009). DPPH antioxidant assay revisited. Food Chem..

[72] Re R., Pellegrini N., Proteggente A., Pannala A., Yang M., Rice-Evans C. (1999). Antioxidant activity applying an improved ABTS radical cation decolorization assay. Free Radic. Biol. Med..

[73] Ko H.J., Kim J., Ahn M., Kim J.H., Lee G.S., Shin T. (2021). Ergothioneine alleviates senescence of fibroblasts induced by UVB damage of keratinocytes via activation of the Nrf2/HO-1 pathway and HSP70 in keratinocytes. Exp. Cell Res..

[74] Kim J., Park S.-H., Yang S., Oh S.W., Kwon K., Park S.J., Yu E., Kim H., Park J.Y., Choi S. (2021). Protective effects of maclurin against benzo[a]pyrene via aryl hydrocarbon receptor and nuclear factor erythroid 2-related factor 2 targeting. Antioxidants.

[75] Hearing V.J. (2000). The melanosome: The perfect model for cellular responses to the environment. Pigment Cell Res..

[76] Park H.Y., Gilchrest B.A. (1999). Signaling pathways mediating melanogenesis. Cell Mol. Biol..

[77] Busca R., Ballotti R. (2000). Cyclic AMP a key messenger in the regulation of skin pigmentation. Pigment Cell Res..

[78] Mitsunaga T., Yamauchi K. (2015). Effect of quercetin derivatives on melanogenesis stimulation of melanoma cells. J. Wood Sci..

[79] Yamauchi K., Mitsunaga T., Batubara I. (2014). Synthesis of quercetin glycosides and their melanogenesis stimulatory activity in B16 melanoma cells. Bioorg. Med. Chem..

[80] Ishida-Yamamoto A., Senshu T., Eady R.A., Takahashi H., Shimizu H., Akiyama M., Iizuka H. (2002). Sequential reorganization of cornified cell keratin filaments involving filaggrin-mediated compaction and keratin 1 deimination. J. Investig. Dermatol..

[81] Kim K.Y., Wang D.-H., Campbell M., Huerta S.B., Shevchenko B., Izumiya C., Izumiya Y. (2015). PRMT4-mediated arginine methylation negatively regulates retinoblastoma tumor suppressor protein and promotes E2F-1 dissociation. Mol. Cell Biol..

[82] Ishikawa Y., Kitamura M. (2000). Anti-apoptotic effect of quercetin: Intervention in the JNK- and ERK-mediated apoptotic pathways. Kidney Int..

[83] Chen T., Zhang X., Zhu G., Liu H., Chen J., Wang Y., He X. (2020). Quercetin inhibits TNF-alpha induced HUVECs apoptosis and inflammation via downregulating NF-kB and AP-1 signaling pathway in vitro. Medicine (Baltimore).

[84] Kim M.Y., Cho J.Y. (2013). 20S-dihydroprotopanaxadiol, a ginsenoside derivative, boosts innate immune responses of monocytes and macrophages. J. Ginseng Res..

[85] Lee J.O., Choi E., Shin K.K., Hong Y.H., Kim H.G., Jeong D., Hossain M.A., Kim H.S., Yi Y.S., Kim D. (2019). Compound K, a ginsenoside metabolite, plays an antiinflammatory role in macrophages by targeting the AKT1-mediated signaling pathway. J. Ginseng Res..

[86] Hossen M.J., Hong Y.D., Baek K.S., Yoo S., Hong Y.H., Kim J.H., Lee J.O., Kim D., Park J., Cho J.Y. (2017). In vitro antioxidative and anti-inflammatory effects of the compound K-rich fraction BIOGF1K, prepared from *Panax ginseng*. J. Ginseng Res..

[87] Han N.R., Ko S.G., Moon P.D., Park H.J. (2021). Ginsenoside Rg3 attenuates skin disorders via down-regulation of MDM2/HIF1α signaling pathway. J. Ginseng Res..

[88] Lee J.Y., Kim C.J. (2010). Arctigenin, a phenylpropanoid dibenzylbutyrolactone lignan, inhibits type I-IV allergic inflammation and pro-inflammatory enzymes. Arch. Pharm. Res..

[89] Kim J.H., Baek E.J., Lee E.J., Yeom M.H., Park J.S., Lee K.W., Kang N.J. (2015). Ginsenoside F1 attenuates hyperpigmentation in B16F10 melanoma cells by inducing dendrite retraction and activating Rho signalling. Exp. Dermatol..

[90] Hong Y.H., Kim D., Nam G., Yoo S., Han S.Y., Jeong S.G., Kim E., Jeong D., Yoon K., Kim S. (2018). Photoaging protective effects of BIOGF1K, a compound-K-rich fraction prepared from *Panax ginseng*. J. Ginseng Res..

[91] Devitt G., Thomas M., Klibanov A.M., Pfeiffer T., Bosch V. (2007). Optimized protocol for the large scale production of HIV pseudovirions by transient transfection of HEK293T cells with linear fully deacylated polyethylenimine. J. Virol. Methods.

[92] Zhang T., Zhong S., Hou L., Wang Y., Xing X., Guan T., Zhang J., Li T. (2020). Computational and experimental characterization of estrogenic activities of 20(S,R)-protopanaxadiol and 20(S,R)-protopanaxatriol. J. Ginseng Res..

